# Training Allied Professionals to Hold Mental Health Support Groups for Children Who Have Experienced Trauma: Can Online Education Meet the Need?

**DOI:** 10.1007/s40596-021-01529-5

**Published:** 2021-09-23

**Authors:** Anna Pérez-Aronsson, Sandra Gupta Löfving, Anna Sarkadi, Georgina Warner

**Affiliations:** Uppsala University, Uppsala, Sweden

In recent years, increased demand has put pressure on child and adolescent psychiatric services in Sweden [1]. A lack of competence and resources to treat trauma is a particular issue, which has been highlighted as problematic for refugees [2]. Thus, efforts have been made to implement a community-based intervention called Teaching Recovery Techniques (TRT) [3] (see Fig. 1 for an overview of TRT). Non-mental health professionals working with children can deliver TRT after a 3-day training [3], regardless of previous knowledge about trauma. The scale-up model is for TRT group leaders who have delivered at least two groups to train further colleagues. Based on a study with mostly male, unaccompanied refugee youth from Afghanistan, TRT has been well received in Sweden and demonstrates promising outcomes [4], consistent with existing evidence that services provided by lay counselors can reduce mental illness [5]. This article will describe an effort to teach TRT to non-mental health professionals remotely via a teleconferencing system.

**Fig. 1:**
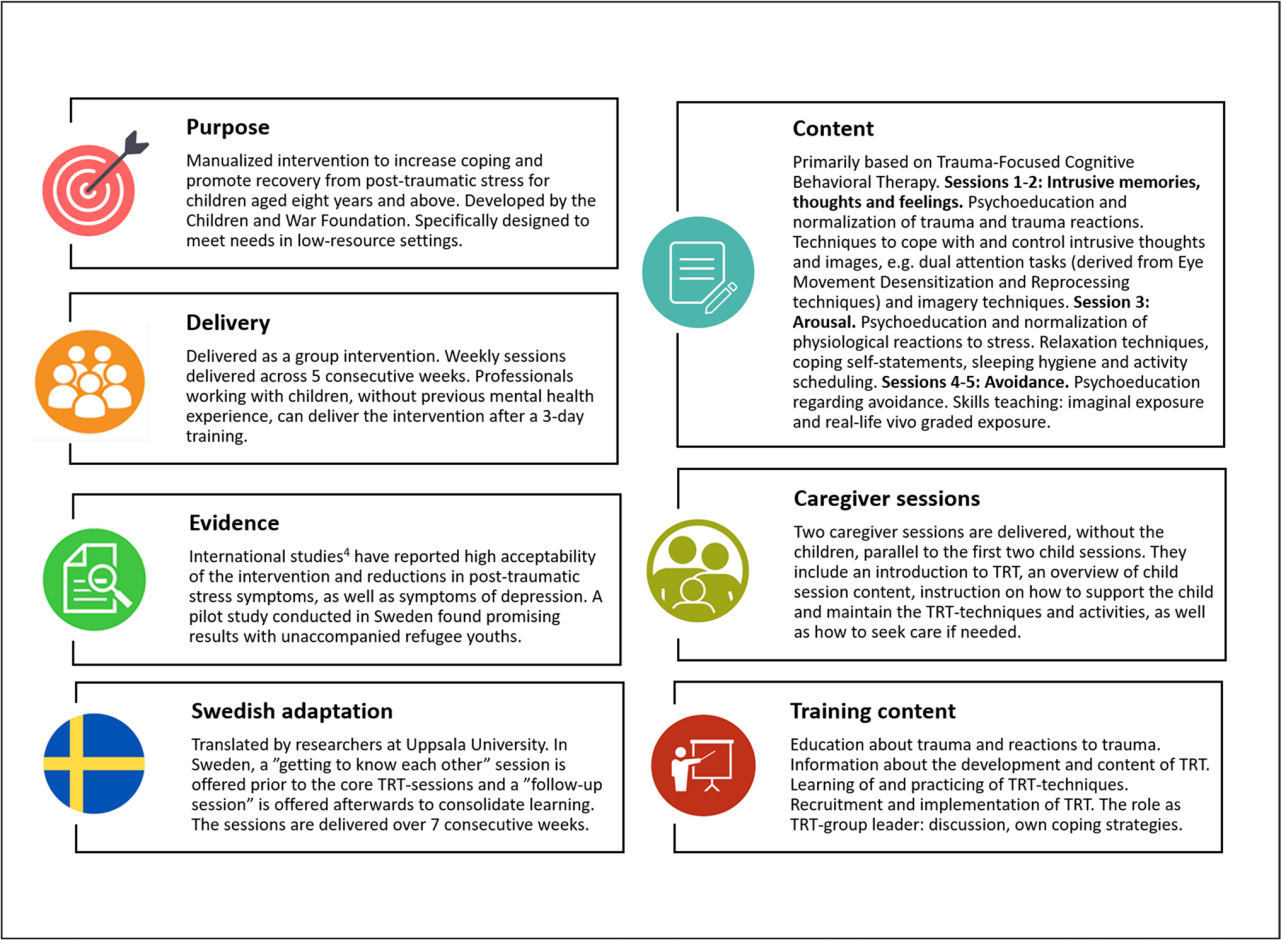
Overview of teaching recovery techniques

In March 2020, COVID-19 motivated the swift decision to conduct a planned TRT training remotely, leaving limited time for preparation. The course included 20 participants from across Sweden and was held by an instructor highly experienced in delivering TRT training, but not online. The same instructor conducted a second online training in June 2020, to which fewer participants signed up (*n* = 5), most likely due to the impact of COVID-19 on occupational tasks. Although the pandemic has increased the amount of online-delivered training, the TRT developers had aspirations to provide remote training prior to the pandemic. There is a perceived need in war-torn areas, such as Syria, where a remote training has been delivered. Yet, to the best of our knowledge, online TRT training has not been evaluated.

The link between training and intervention fidelity is known [6] and is important among professionals leading interventions outside their typical area of expertise [7]. Research suggests that online professional development is feasible [8]. However, successfulness might vary depending on the suitability of content for online training [9]. Training lay counselors online may require different implementation strategies than for mental health professionals, especially with certain high-risk populations [10]. A prevailing need in the context of trauma intervention is for professionals without previous psychiatric training to be adequately equipped to cope with challenges such as suicidal ideation [11]. This study adds to the field by exploring the professionals’ own experiences of online-delivered TRT training, which can guide recommendations for future online trauma-support training.

## Interviewing Course Participants

Nine individual videoconference interviews were conducted using a semi-structured interview guide. The guide was based on the Kirkpatrick 4-level model [12] and assessed in a pilot interview. The Swedish legislation on ethical review for research involving humans (SFS 2003:460) applies to research that involves processing personal sensitive data, which does not include interviews with professionals about their work tasks. This research could therefore be conducted without applying for ethical review from the Swedish Ethical Review Board, but in accordance with the Helsinki Declaration. All participants received information about the study that participation was voluntary and that no identifying details would be reported.

All attendees from the two online TRT trainings (*N* = 25) were contacted via e-mail and a social media group, asking those who were interested in participating to contact the researchers. The interview participants were all female, aged 40–60 years, working with children (4 school counselors, 2 teachers, 1 social educator, 1 student ombudsman, 1 social worker), reflecting the demographic of the wider training attendees. The interviews, lasting an average of 30–min, were recorded, transcribed, and analyzed using systematic text condensation [13]. Dependability was established by cross-checking: three coauthors independently read all transcripts to reach a comprehension of the content and checked the coding process. Then, all coauthors discussed the results to agree on themes and codes and validate the identified themes. Initial analysis took place after the first seven interviews, which were conducted by the first author in April 2020. The second author conducted two more interviews in September 2020. All authors agreed that these later interviews contained the same themes as the previous and that no new themes emerged. Thus, it was decided that no further data collection was required. The data were securely stored, and the analysis procedure was well documented to enable the research process to be followed, audited, and critiqued to ensure dependability and confirmability. The findings guided practical recommendations, shared back with training participants for validation in a brief, anonymous Web survey.

## Experiences of Online Training

Analysis resulted in two main categories (Table 1). “Learning TRT together online” was formed by participants’ description of online training as a positive experience, mainly thanks to interactivity. “Self-perceived learning and need of support” captured participants’ increased confidence in helping children with trauma-related symptoms and their remaining support needs.

**Table 1:** Overview of the results and related recommendations for future online training

Main categories	Learning Teaching Recovery Techniques (TRT) together online			Self-perceived learning and need of support	
	Subcategories				
	Increased accessibility and sustainability	An interactive learning environment	The importance of structure, mindset, and experience	Increased knowledge and confidence in encounters with children who have experienced trauma	Holding a TRT group: hopes and support needs
Example of quotes	“I live in a sparsely populated county, where it is tricky with communications /…/ and I’ve noticed that there is a lot that is no available to us. It takes so much for to be able to participate, which makes it difficult to get employers onboard /…/ It is travel and accommodation that makes [it costly]” (Interview 3)	“I imagine before the course that it would be a lot of physical exercise, and that you would have to try using the tools a lot. And I was surprised that it worked so well to do that online. I didn’t think the effect of those parts were necessarily lost” (Interview 1)	“Quite high demands are placed on the person holding the course. When it comes to allocating turns to speak, and keeping time for breaks, and being clear and keeping the commitment up. When you’re just sitting and looking at a screen it demands quite a lot of the person who is speaking” (Interview 5)	“I think what I primarily take with me is an increased understanding of what trauma is, and how trauma can look. And that there is a toolbox, which needn’t be so complicated. That you can with small means help someone who has been in trauma” (Interview 6)	“It feels like a pretty big challenge, a lot to keep track /…/ At the same time, the needs are great and this is a great support, so I absolutely want to do this /…/ It would be good with an online network for everyone who has been TRT-trained during these years, so we can become more people who can collaborate and support each other” (Interview 7)
Implicated recommendations for future online TRT training	➢ **Prior to training, send out:** A printed manual Instructions for preparatory reading and videos Advice on how to prepare the physical environment for practicing techniques Physical aids (e.g., essential oils) to assist with practicing techniques Advice on technical requirements for the online course ➢ **During the training:** Take regular short breaks Teacher-to-participant ratio of 1:6 Teacher assigned to receiving queries (e.g. via “chat”) in larger group sessions Several opportunities for facilitated small-group discussions Utilization of visual aids throughout			➢ **During the training:** Several opportunities for facilitated small-group discussion Introduction of an experienced TRT facilitator as a “mentor” to each small group ➢ **After the training:** Offer a booster session Facilitation of “keeping in touch” with small group members and mentor Offer participation in a larger TRT network (but not compulsory)	

## Learning TRT Together Online

### Increased Accessibility and Sustainability

Online training was described as cost saving and supporting a reduced carbon footprint. Increased accessibility was particularly important for remote and/or low-resource settings. Employers and regional decision makers in remote regions were described as “forgetting” that children with trauma-related symptoms exist outside of cities, as a consequence of the lack of trauma interventions: “…you miss [trauma-related symptoms] because we can’t do anything, anyway. If the possibilities to offer support increases, I think awareness would increase too” (Interview 3).

### An Interactive Learning Environment

The interactions exceeded expectations: “I was surprised that the course held pretty high quality. That the interactions worked so well. I felt like I was participating, that I was part of the group” (Interview 5). Playing games and reflecting on the material together in smaller groups was seen as valuable for group cohesion and learning. Participants would have appreciated more chances to discuss course content, especially since training online meant missing opportunities to socialize, such as during lunch.

The bigger group used the chat to ask questions without interrupting, which was perceived as useful but potentially stressful for the teacher: “You may need to be two [teachers]. To keep up with questions in the chat. She was very good at it, but it must have been very strenuous” (Interview 6). The lower number of participants in the second training was believed to facilitate learning; “If you’re 6, it is perfectly ok to interrupt with a question, but if you are 10 to 15 it becomes much more difficult” (Interview 9).

Participants had expected on-site training to involve active practicing of techniques and were positively surprised this was feasible online. Activities involving the body (e.g., the “dual-attention task,” which involves clapping your hands on your knees while thinking of a difficult memory), were perceived as better suited for on-site training due to the restricted view of the instructor and other attendees on screen. Other techniques (e.g., imagery techniques) were thought to be easier online as physical distance might decrease self-awareness.

The teaching methods had been varied, which had been important to maintain focus online. The participants gave suggestions to further increase learning, such as more visual aids, having the physical TRT manual, and receiving instructions beforehand on how to prepare for practicing techniques.

### The Importance of Structure, Mindset, and Experience

Online TRT training was perceived to demand more than on-site in terms of concentration, flexibility, structure, and previous experience: “I thought about those [participants] who have never heard of it [trauma] /…/ I’m pretty familiar with it. And yet it took quite a lot of energy to sit like this in front of the computer. So it’s great that she [the teacher] put in many short breaks” (Interview 4). Group discussion was regarded as important for learning and to enable future peer support, the absence of which was considered particularly detrimental to participants without previous experience.

Similarly, the teacher’s experience of TRT was seen as fundamental: “I thought it would be trickier to try the techniques and learn them. But no /…/ that probably depended very much on the teacher’s ability to convey it so pedagogically and methodically, as she is so comfortable with the manual” (Interview 1).

## Self-Perceived Learning and Need of Support

### Increased Knowledge and Confidence in Encounters with Children Who Have Experienced Trauma

The online course met the participants’ needs; they felt more confident to work with children who had experienced trauma: “I work a lot with decreasing the fear of trauma. It’s so charged, you’re so scared to make a mistake /…/ knowing that there is a method kind of discharges a lot of what feels so charged. Because we could actually offer something here in our municipality” (Interview 3). Yet, a need was expressed to rehearse the manual. Those from the latter training had received an appreciated booster session (an independent decision of the training provider): “It was super good. Even if it wasn’t new, some questions had come up and you get this reminder” (Interview 8).

### Holding a TRT Group: Hopes and Support Needs

Participants were eager to hold a TRT group, seeing a clear need among the children they met. Yet, they also voiced insecurities: “It feels like a pretty big challenge, a lot to keep track of /…/ But the needs are great and this is a great support, so I definitely want to do this” (Interview 7). One participant was holding a TRT group, and others described that they had wanted to, or even planned to, but had not been able due to social restrictions during the pandemic or lacking the required facilitation partner. A supportive structure at the workplace and support from other group leaders seemed to increase confidence about holding groups. However, online training was believed to make it less likely that participants stayed in touch: “It can be good to have these contacts, to exchange experiences /…/ to give each other good advice. And those contacts may not be made naturally because you don’t talk to each other during the breaks, you are only active during the training itself. So that’s probably what you miss [when training online], so I think you might need to have some plan for that in advance” (Interview 2).

### Recommendations for Future Online Training

Based on the findings of the interviews, recommendations for future online TRT training were developed and presented back to the participants in a Web-based, anonymous survey after the interviews were concluded. Table 1 shows the interview findings and the corresponding recommendations endorsed by participants.

This study has various limitations. Participants volunteered, which could create selection bias. They were mostly female school staff, but this reflects the real-world intervention setting. It was a small-scale qualitative evaluation; thus, the results cannot be expected to be generalizable to an international context or all online professional training. However, data saturation was achieved, and the participants worked in different geographical areas in Sweden and had different background knowledge of trauma and group support, which can promote transferability. Cross-checking (i.e., three authors going through all transcripts and coding) was used to achieve dependability and confirmability.

## Implications for Practice

The participants described increased confidence to support children who have experienced trauma, echoing previous findings that training community professionals can lead to empowerment and better understanding of mental health issues [5]. Overall, they were satisfied that online training had met their needs. Thus, online TRT training could be a feasible and acceptable alternative, which could increase intervention availability. The resultant recommendations address important needs and can facilitate meaningful training of lay counselors. Although many of the recommendations could be applied to several online training programs, one could argue that some hold greater value in the context of trauma intervention training. For example, having an experienced mentor could overcome the known “fear” of certain trauma therapy techniques, such as exposure therapy [14].

Interaction was highly valued, aligning with the social constructivist perspective asserting that learning is formed through social processes such as conversation [15], as well as previous research on online professional training showing that interactive training increases participants’ involvement and enjoyment [8, 10]. This need for meaningful interaction underpinned a recommendation for smaller training cohorts. Yet, according to the post-interview survey, participants did not find the recommendation favorable. This could be due to the perceived need for “critical mass,” whereby there are enough participants for the training to be meaningful [16]. An alternative recommendation is larger training cohorts but a good teacher-to-participant ratio (e.g., 1:6) to enable discussions in intimate groups throughout the program while having access to a teacher to answer questions, which has been found to be valued in online professional training [8].

Insecurities about holding a TRT group echo previous implementation research and thus should not be attributed to the online format. For instance, organization-level characteristics and post-training support, identified as important in this study, also significantly predicted the use of a community-based parenting program [17]. Moreover, networks have been highlighted as important for the implementation and maintenance of TRT in Sweden [18] and it has been shown that allied professionals benefit from continuous supervision, particularly in remote/rural areas [19] and after brief training [10]. When conducting online mental health training of allied professionals, the need to build networks and communities of practice should be considered.

This study suggests the online format might be more challenging for professionals without previous trauma training. This differs from a recent study evaluating an online teacher education in trauma-informed care, which reported all participants experienced enhanced performance on attitudes towards trauma and self-efficacy, regardless of previous training [20]. Yet, this education took place over several weeks and included an online discussion forum, which could benefit less experienced attendees. Thus, a larger-scale evaluation of online TRT training, preferably utilizing quantitative evaluation forms, should further explore if the online format is a disadvantage for professionals without previous trauma training and, if so, how this disadvantage can be addressed.
